# RadLex and SNOMED CT integration: a pilot study for standardising radiology classification

**DOI:** 10.1186/s13244-025-01935-5

**Published:** 2025-03-13

**Authors:** Merit Marquis, Igor Bossenko, Peeter Ross

**Affiliations:** Tallinn University of Technology, Tallinn, Estonia

**Keywords:** Radiology, Interoperability, Data capture, SNOMED, LOINC

## Abstract

**Background:**

Effective communication and information exchange across diverse platforms are critical in healthcare data systems. However, the presence of multiple coding systems and varying standards creates discrepancies and misalignments, highlighting the need for innovative solutions to address these challenges.

**Objective:**

The study aimed to develop a technical and semantic interoperability method specifically for radiology procedures, utilising the terminology management tool TermX to facilitate efficient data exchange and utilisation in healthcare.

**Results:**

The study resulted in a revised RadLex data model using SNOMED CT, accompanied by a mapping guide and a classification system for X-ray and angiography procedures. This classification system consists of nineteen distinct properties, each defined by specific value sets derived from SNOMED CT terminology. A total of 380 concepts were utilised to describe the 622 procedures examined comprehensively.

**Conclusion:**

Through twelve design cycles involving in-depth analysis and iterative refinement, the mapping of angiography and X-ray procedures was successfully achieved, culminating in the creation and validation of a universal model that enhances both primary and secondary data collection. The efficacy and innovation of this system pave the way for further advancements in healthcare interoperability.

**Critical relevance statement:**

The innovative integration achieved in this study for standardising radiology classification promises to improve data management practices and enhance patient care outcomes through increased interoperability within the healthcare sector.

**Key Points:**

- A universal radiology procedure model to enhance capture would be valuable.
- A tool to facilitate technical and semantic interoperability for efficient data exchange in healthcare was created.
- This system could pave the way for futher advancements in healthcare interoperability.

**Graphical Abstract:**

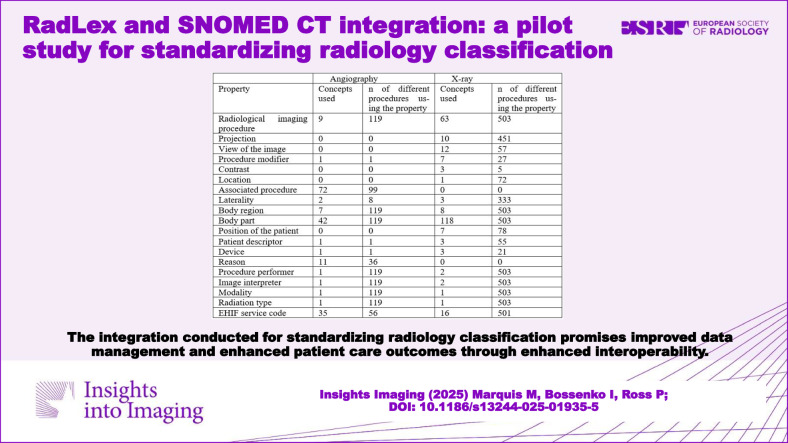

## Introduction

Healthcare standardisation has three primary purposes: organising clinical concepts, enabling accurate billing, and providing public health insights [1]. However, the use of diverse coding systems across healthcare domains gives rise to interoperability issues, presenting a global challenge.

Global dependence on varied medical coding systems has led to significant interoperability issues. Many coding systems were established without considering ontological developments, hindering data integration and information exchange. Countries such as Denmark, Sweden, Albania, and Indonesia encounter these challenges, which affect health information sharing and technology standardisation [2–5]. Despite technological progress, achieving semantic interoperability remains difficult, indicating the need for an effective translation platform [6, 7]. This study aims to create a method for technical and semantic interoperability in radiology, using the terminology management tool TermX to develop a classifier system that enhances data exchange and use. TermX serves as an open-source platform that promotes interoperability through knowledge sharing [8–13].

The study aimed to improve primary data use and radiology procedure ordering by integrating various coding systems. The resultant classifier system also supports secondary data applications. A standardised classifier system is essential for facilitating data analysis, enabling effective data utilisation for research, policy, and quality improvement.

The paper consists of four chapters. Chapter One introduces the terminologies used and the issues in the classifiers of Estonia’s radiology procedures and billing codes. Chapter Two presents the study’s methodology, outlining the approaches and tools used. Chapter Three presents the results in different subchapters: modifications to local term mapping guidelines, method cycles with an explanatory table, the classifier system data model structure, and the mapping of radiological procedures. Chapter Four discusses the iterative development process and challenges in creating a unified coding framework, emphasising the integration of SNOMED CT with RadLex to improve mapping accuracy, particularly for angiography (ANG) and X-ray procedures. The chapter discusses the significance of incorporating contextual properties in the coding schema for better data interpretation, efficiency in health information exchange, and operational improvements in healthcare settings. It also examines the broader implications of these findings, including the benefits for healthcare organisations, administrative efficiency, radiology departments, and patient outcomes, as well as their potential to inform public health initiatives and resource allocation.

### Terminologies of data exchange

Semantic interoperability, which is the ability to exchange and understand the context of data across diverse systems consistently, relies on terminologies, classifications, and coding systems to organise and access information correctly. Standardised terminologies provide essential clinical terms that form the backbone of classification systems used to document patient information [14]. Classification systems further categorise entities based on shared attributes, creating organised groupings that facilitate easier access and analysis [15]. Coding systems build on these classifications by assigning unique identifiers to entities, enabling efficient information organisation and analysis [16].

However, achieving this interoperability becomes complex when integrating multiple coding systems [17, 18]. Differences in standards, terminologies, and data formats across systems present considerable interoperability risks. These discrepancies can lead to potential delays in treatment, duplicate medical reports, and even errors in statistics or clinical data specification [18, 19].

LOINC (Logical Observation Identifiers Names and Codes), a universal system for laboratory tests, has been extended to other clinical areas such as radiology. A collaboration between LOINC and the Radiological Society of North America (RSNA) known as RadLex (Radiology Lexicon), presents a comprehensive radiology terminology system that serves various purposes such as reporting and decision support [20]. However, integration into a local code system necessitates a mapping process that, particularly with LOINC codes, is laborious, time-consuming, often impeded by language barriers, and requires expert knowledge for accurate mapping [21].

#### SNOMED CT

SNOMED CT (Systematised Nomenclature of Medicine Clinical Terms) the largest clinical terminology system with approximately 370,000 concepts, was developed to ensure the consistency and processability of clinical information in electronic health records [22]. This standardisation plays a critical role in decreasing diagnostic errors and enhancing the inclusiveness of reports by ensuring uniform usage and understanding of relevant anatomical and clinical terms across diverse healthcare settings [23]. SNOMED CT is being used by 49 countries [24].

### Challenges in achieving interoperability in radiology

#### Classifier of radiology procedures

Over the past 25 years, the Estonian Society of Radiology (ESR) has developed a classifier for radiological procedures, organised into three hierarchies: modality, body region, and anatomic focus (Appendix 1 and Table 1) [25]. Despite this effort, the classifier suffers from structural incoherence, which presents significant challenges to achieving interoperability. Ambiguous mappings between procedure codes and billing codes, inconsistent naming conventions, and the inclusion of surgical procedures within radiological codes all contribute to confusion and errors in data interpretation. For example, some procedure codes reference multiple procedures under one code or split a single procedure into separate codes, leading to mismatches in data exchange. This lack of standardisation complicates the integration of data between systems, impeding the efficient sharing, analysis, and reporting of radiological data. Furthermore, as the names of many procedures are poorly defined or misaligned, it becomes difficult to align them accurately with other coding systems, hindering effective communication and data sharing between organisations.

#### Code list for billing healthcare services

Estonia’s healthcare system relies on a unified universal health insurance system, managed by the Estonian Health Insurance Fund (EHIF), which is responsible for healthcare financing and the reimbursement of services [26, 27]. However, the coding system used by the EHIF for billing purposes creates another barrier to interoperability. While the EHIF’s codes are designed to manage reimbursement and financial support, they do not always align with the procedure codes used by the ESR (Appendix 1 and Table 2). This misalignment occurs because the EHIF list is focused primarily on reimbursed services, whereas the ESR’s coding system encompasses a broader range of radiological procedures. As a result, when radiological procedures are mapped between these two coding systems, discrepancies arise, leading to issues in the transfer of data and preventing the accurate mapping of procedures between systems. These inconsistencies hinder interoperability, making it challenging to exchange and reconcile data for both financial and clinical purposes.

#### Data exchange of code lists

The Estonian Health and Welfare Information Systems Centre (TEHIK) manages the national Health Information System, which serves as a central hub for data exchange between patients, healthcare providers, the EHIF, and medical specialities such as the ESR [28]. While TEHIK plays a crucial role in facilitating the exchange of health data across the country, it does not offer services to standardise, harmonise, or correct the classifiers developed by medical specialities. This results in a fragmented system where multiple, structurally different code lists are used across the healthcare sector. These inconsistencies in data formatting and classification increase the risk of interoperability errors as healthcare entities struggle to reconcile different standards and frameworks. The lack of a unified approach to standardising medical data across specialities further exacerbates issues in achieving seamless, accurate data exchange between systems.

## Methods

For this study, the design science research (DSR) methodology was employed, which is a structured approach designed to tackle real-world problems through iterative, problem-driven research. The methodology encompasses several phases with cycles, including detailed design, implementation, real-world testing, reflection on outcomes, results validation, and documentation of the process, ensuring that practical and effective solutions are achieved [29]. The ESR coding system for radiology procedures was obtained from the TEHIK publishing centre [25]. An initial examination of the list revealed 2,341 procedures across 11 distinct modalities. For a comprehensive analysis, two specific modalities were selected: ANG (*n* = 119), identified as the most complex modality to describe and map due to its association with surgical procedures, and X-ray (*n* = 503), recognised as the most widely utilised diagnostic imaging technique. The structural data model was based on RadLex [30], and a nine-step mapping guideline from SNOMED CT for local terms was utilised [31]. Given the absence of translations for certain concepts in the SNOMED CT Estonian Edition, the International Edition from 31 July 2023 was employed. TermX was chosen as the terminology management platform, leveraging medical dictionaries and lexicons for concept translations [10]. Formal definitions were established within the coding system to ensure uniqueness. TermX validated these formal definitions to prevent duplication of procedures based on identical property sets.

## Results

### DSR cycles

This study consisted of a total of twelve iterative cycles of DSR, focusing on problem-solution development. Each cycle was informed by the findings of the preceding cycle, enabling the identification and resolution of obstacles encountered during the analysis and mapping phases. Of these twelve cycles, three were categorised as ‘failures’, one cycle successfully validated a hypothesis, and eight cycles were deemed successful. A more detailed description of the steps taken throughout these cycles and their results can be found in Appendix 1 and Table 3.

### Guideline for mapping local codes to SNOMED CT

A mapping guideline for utilising SNOMED CT to map local terms was employed when it became evident that Radlex/LOINC cannot support surgical names and needs to be exchanged for SNOMED CT; however, due to the non-coherent structure of the ESR coding system, the necessary adjustments to the original mapping guideline were implemented [31]. A summary of these adjustments can be found in Appendix 2 and Fig. 1.

### Data model

The classification system for ANG and X-ray procedures in TermX has 19 properties which are categorised into three groups [32]. All properties have their own corresponding value set, except ‘Procedure performer’ and ‘Image interpreter’, which share one value set. Each value set was flat with no hierarchy and had no overlapping concepts between value sets. The classifier includes 380 distinct SNOMED CT concepts needed to describe all 622 procedures (Appendix 1 and Table 4).

### Angiography and X-ray

In ANG (Appendix 1 and Table 5), all 119 distinct procedures were structured utilising a maximum of 14 properties. More than three-quarters (*n* = 99) necessitated the inclusion of an ‘associated procedure’ property, with the primary focus being surgical procedure. The property ‘reason’ was required 35 times to enhance the differentiation between similar procedures. A total of 35 distinct EHIF service codes were employed across 56 procedures, resulting in a success rate of 47%.

In X-ray (Appendix 1 and Table 5), all 503 procedures were structured utilising 17 properties. No X-ray procedure necessitated the inclusion of an ‘associated procedure’ or ‘reason’ property. The primary source of differentiation among procedures stemmed from the combination of the ‘radiological imaging procedure’ (*n* = 63) and the ‘body part’ (*n* = 118). A total of 501 procedures and 16 diverse EHIF service codes were employed, resulting in a success rate of 99.6%.

## Discussion

Iterative DSR cycles revealed the intricate challenges of developing a unified coding framework. Although RadLex with LOINC seemed initially suitable, its inadequacy in representing ANG procedures highlighted the need for a more flexible system. Incorporating SNOMED CT into the RadLex model markedly improved mapping efficacy, facilitating better representation of ANG and X-ray procedures. Integrating contextual properties within the coding schema was vital for a better understanding of procedures, which is essential for statistical evaluation. This transition not only enhances data interpretation accuracy but also fortifies the dependability of health information exchange among providers. TermX, which incorporated 19 unique properties for ontology development, demonstrated flexibility and efficacy, signifying its potential utility for healthcare organisations aiming to refine coding practices, even with limited ontology expertise. The implications of these findings transcend theoretical frameworks, influencing multiple healthcare sectors with improved primary and secondary data collection. For health funds, precise procedure representation enhances billing accuracy, minimising the necessity for manual clarifications and thereby optimising administrative efficiency. Differentiating procedures based on complexity or cost supports thorough data analysis, aiding in identifying usage patterns and cost determinants for informed budgeting. In primary care, improved clarity and accuracy enhance the ordering workflow, enabling providers to efficiently select suitable radiology procedures based on their properties with reduced manual input. This advancement mitigates data entry inaccuracies and promotes integration with clinical decision support systems, ultimately improving decision-making and patient outcomes. Within radiology departments, these findings promote the clearer identification of the required procedures, reducing administrative load and improving operational efficiency. By allocating complex procedures to specialists, resource utilisation and operational effectiveness are optimised. Accurate documentation of procedure types, complexities, and outcomes improves internal quality assurance, benefitting patient care. Clarifying procedure descriptions enhances collaboration between radiologists and general practitioners, reducing the need for further clarifications or corrections, thereby accelerating service delivery, a crucial aspect of enhancing healthcare experiences. The advantages extend to patients via precise referral letters that accurately outline procedures, diminishing the chances of return visits. Assigning specialists based on procedural necessities encourages personalised treatment strategies and improves outcomes. Streamlined workflows and accurate documentation not only reduce delays in radiology services but also enable quicker diagnosis and treatment, especially in urgent cases where timely intervention is critical. From a public health viewpoint, the study provides structured and reliable data for evaluating trends, resource distribution, disease prevalence, and the public health ramifications of radiological procedures. Making informed healthcare resource decisions is crucial; identifying prevalent procedures, monitoring demand fluctuations, and recognising service deficiencies can inform future healthcare initiatives. Moreover, this data allows for the tracking of disease occurrences necessitating radiological procedures and assesses disparities in diagnostic service availability across different regions and demographics.

## Conclusion

The developed model significantly enhances data collection and resolves interoperability challenges in healthcare, especially in radiology. Using the TermX terminology management tool, the system creates a coherent coding framework that improves procedure accuracy and streamlines ordering and billing. Contextual properties are incorporated to clarify and specify procedure complexities. This universal model addresses issues faced by countries with self-made coding systems that lack terminological evolution. Its implementation enhances primary and secondary data collection internationally. Standardised coding enhances primary data accuracy, reducing patient record errors and improving health information reliability. Improved secondary data collection facilitates clearer health analytics for healthcare organisations. This leads to informed resource allocation decisions and better healthcare budgeting, enhancing service management. By enabling precise reporting on procedures, the model supports internal quality control and patient care improvement in radiology departments. The generation of reliable data benefits public health agencies in assessing disease prevalence and monitoring healthcare trends. This improved data environment aids strategic planning at the population level. The interoperable coding framework promises a paradigm shift in health information exchange, enhancing patient care quality and healthcare system efficiency globally.

## Supplementary information

ELECTRONIC SUPPLEMENTARY MATERIAL

## Data Availability

The classification system created during the current study is available in the “ee-radiology-services” repository, https://github.com/termx-health/ee-radiology-services.

## Abbreviations

ANG: Angiography
DSR: Design science research
EHIF: Estonian Health Insurance Fund
ESR: Estonian Society of Radiology
LOINC: Logical Observation Identifiers Names and Codes
RadLex: Radiology Lexicon
SNOMED CT: Systematised Nomenclature of Medicine Clinical Terms
TEHIK: Estonian Health and Welfare Information Systems Centre
