# Supplementary material for: RadLex and SNOMED CT integration: a pilot study for standardising radiology classification
Source: Insights Imaging. 2025 Mar 13;16:58. doi: 10.1186/s13244-025-01935-5 (PMC11906921; doi:10.1186/s13244-025-01935-5)

RadLex and SNOMED CT Integration: A Pilot Study for Standardising Radiology Classification

ELECTRONIC SUPPLEMENTARY MATERIAL

Appendix 1 - Tables

Table 1. Example of the ESR radiology coding system

Modality	Body region	Anatomic focus	Procedure name	Code
ANG[1]	AJ[2]	Arteries	Lower limb arteries, right	ANGAJ001
ISU[3]	KA[4]	Thyroid	99mTc-pertechnetate thyroid scintigraphy	ISUKA001
OPT[5]	PE[6]	Skull	X-ray of the bones of the facial skull (lateral view)	OPTPE003
RGU[7]	RK[8]	Ribs	X-Ray of ribs, (oblique) left and right	RGURK021

[1] Angiography; [2] Lower limb; [3] Isotope procedures; [4] Neck; [5] Orthopantomography; [6] Head; [7] X-ray; [8] Chest

Table 2. Comparison of procedure codes in EHIF and ESR coding system

Procedure name	EHIF code	ESR code
X-Ray of ribs, (oblique) left and right	7904	RGURK021
X-Ray chest region (two images)	7903	N/A
X-Ray chest region (three or more images)	7904	N/A
Endocardial mapping	7658	ANGRK014
Endocardial mapping. Treatment of arteriovenous malformation	6108	ANGRK033
Closure of patent ductus arteriosus	N/A	ANGRK007
Renal artery stenting	N/A	ANGKV013

Table 3. Flow of DSR cycles

Description	Activity	Result	Reason
Cycle 1: Initial analysis of radiology procedure information and RadLex/LOINC capability.	Analysed 10 angiography (ANG) procedures to assess RadLex/LOINC model compatibility.	Failure	ANG procedures often included surgical components, which were not fully represented by RadLex alone, highlighting the need for a more adaptable mapping approach.
Cycle 2: Addition of SNOMED CT to the RadLex/LOINC to support surgical annotation in the mapping process.	Attempted mapping of 10 ANG procedures using RadLex/LOINC model combined with SNOMED CT concepts.	Failure	Integration of SNOMED CT led to code duplications across procedures, complicating the mapping process and indicating a need to reconsider the combined use of RadLex, LOINC, and SNOMED CT.
Cycle 3: Replacing LOINC with SNOMED CT and adding custom properties to RadLex to support surgical annotations	Mapped 10 ANG procedures using RadLex model and SNOMED CT concepts, incorporating additional custom properties to capture specific details.	Success	The added properties helped overcome the limitations previously encountered limitations, enabling clearer, more accurate mapping without LOINC duplication.
Cycle 4: Full mapping of ANG procedures	Mapped all remaining ANG procedures with the refined RadLex/SNOMED CT model and added properties.	Success	The custom properties allowed for total mapping of ANG procedures, demonstrating the new model's capacity for accurate representation with only RadLex and SNOMED CT.
Cycle 5: Initial trial mapping of X-Ray (XR) Procedures	Mapped 10 XR procedures with RadLex model and SNOMED CT concepts.	Success	Although successful, this trial revealed the need for additional properties to better capture specific XR data, setting the groundwork for further model enhancement.
Cycle 6: Identification and addition of new properties for XR procedures	Mapped all XR procedures, adding properties to RadLex/SNOMED CT to accommodate unique XR details.	Success	The additional properties extended the model's scalability, allowing it to handle a broader spectrum of XR information accurately.

**Cycle 7: Re-evaluation and addition of properties for non-stated Information**	**Re-mapped all ANG and XR procedures with RadLex/SNOMED CT, incorporating new properties for enhanced data capture.**	**Success**	**The updated model proved scalable, successfully managing expanded data requirements for both ANG and XR procedures.**
**Cycle 8: Trial mapping of XR procedures to the EHIF code system by creating a new property corresponding to the EHIF code**	**Mapped 10 XR procedures, testing the inclusion of the EHIF code system.**	**Success**	**Adding the EHIF property allowed procedure names from ESR and EHIF to align, confirming the model's semantic compatibility.**
**Cycle 9: Complete mapping of XR procedures with the new EHIF property**	**Mapped all remaining XR procedures with the EHIF property.**	**Success**	**Consistency across XR procedure names from both systems validated the model's capacity for semantic alignment.**
**Cycle 10: Trial mapping of ANG procedures with EHIF property**	**Mapped 10 ANG procedures, incorporating the EHIF property to test system compatibility.**	**Failure**	**ANG procedures included surgical aspects not represented by EHIF, causing semantic misalignment between ESR and EHIF standards.**
**Cycle 11: Validation of interoperability issues between ESR and EHIF systems**	**Remapped all ANG procedures with the EHIF property to confirm interoperability challenges.**	**Proven valid**	**The need for improved terminology alignment was validated, as ANG procedures retained surgical descriptions that conflicted across coding systems.**
**Cycle 12: Final validation and publishing**	**Conducted TermX automatic validation and finalised the model for publication.**	**Success**	**The completed system met interoperability requirements with unique codes readable by both humans and machines, excluding ANG and EHIF alignment, concluding the study with a scalable and universally applicable model.**

Table 4. Description of the data model

Group	Property	Description	Value-set	Top-hierarchy	n of unique concepts
Related to radiological imaging	Radiological imaging procedure	Imaging procedure	imaging-procedure	363679005 |Imaging (procedure)|	72
	Projection	Direction of the beam	projection	260419006 |Projection (qualifier value)|	10
	View of the image	Imaging perspective	view	309825002 |Spatial and relational concepts (qualifier value)|	12
	Procedure modifier	Modification to the imaging procedure	procedure-modifier	272099008 |Descriptor (qualifier value)|	8
	Contrast	Contrast or substance used	pharmaceutical	763158003 |Medicinal product (product)|	2
	Location	Location of the procedure	location	285201006 |Hospital environment (environment)|	1
	Associated non-radiological procedure	Associated procedure to the imaging procedure	associated-procedure	128927009 |Procedure by method (procedure)|	72
Related to patient	Laterality	Body side	laterality	106233006 |Topographical modifier (qualifier value)|	3
	Body region	Body region	body-region	91723000 |Anatomical structure (body structure)|	9
	Body part	Body part	anatomic-focus	52530000 |Body region structure (body structure)|	159
	Position of patient	Position of the patient	body-position	9851009 |Body position finding (finding)|	7

	**Patient descriptor**	**Patient profile based on age/sex/gravid**	**person**	**410598002 |Person categorized by age (person)|**	**4**
	**Device**	**Medical device**	**device**	**63653004 |Biomedical device (physical object)|**	**4**
	**Reason**	**Indication for the procedure**	**reason-for-exam**	**64572001 |Disease (disorder)|**	**11**
**General**	**Procedure performer**	**Performer of the procedure**	**speciality**	**223366009 |Healthcare professional (occupation)|**	**4**
	**Image interpreter**	**Interpreter of the image**			
	**Modality**	**Imaging method**	**modality**	**363679005 |Imaging (procedure)|**	**2**
	**Radiation type**	**Radiation type used**	**radiation**	**82107009 |Radiation (physical force)|**	**1**
	**EHIF service code**	**EHIF healthcare service code**	**ttl**	**N/A**	**N/A**

Table 5. Mapping results of angiography and X-ray

	Angiography		X-ray	
Property	Concepts used	n of different procedures using the property	Concepts used	n of different procedures using the property
Radiological imaging procedure	9	119	63	503
Projection	0	0	10	451
View of the image	0	0	12	57
Procedure modifier	1	1	7	27
Contrast	0	0	3	5
Location	0	0	1	72
Associated procedure	72	99	0	0
Laterality	2	8	3	333
Body region	7	119	8	503
Body part	42	119	118	503
Position of the patient	0	0	7	78
Patient descriptor	1	1	3	55
Device	1	1	3	21
Reason	11	36	0	0
Procedure performer	1	119	2	503
Image interpreter	1	119	2	503
Modality	1	119	1	503
Radiation type	1	119	1	503
EHIF service code	35	56	16	501

Appendix 2 – Figures

Figure 1. Overview of modifications to the mapping guideline

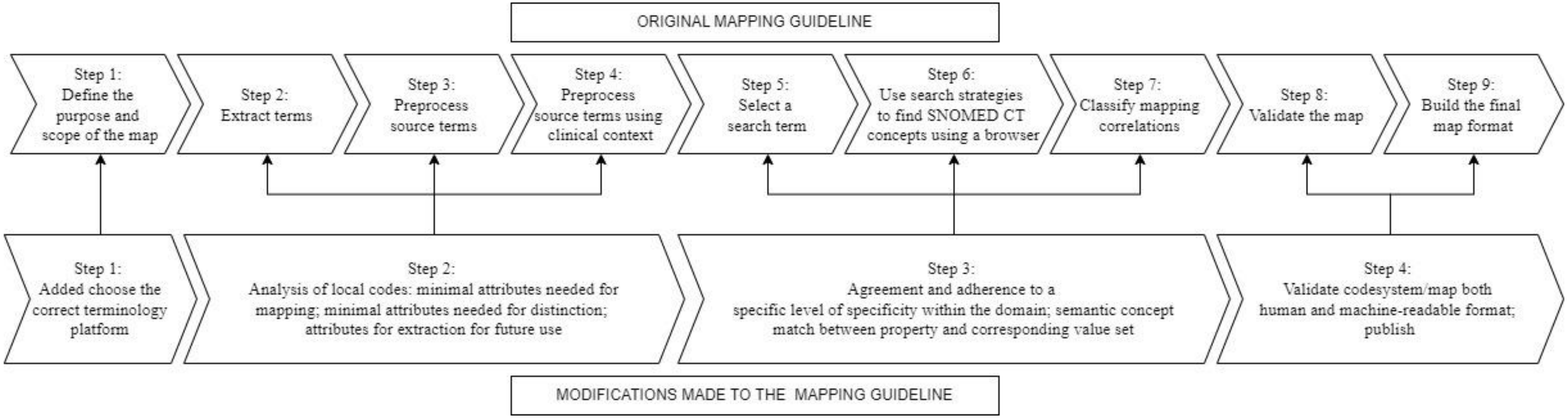

Supplement: Supplementary file 1 — ELECTRONIC SUPPLEMENTARY MATERIAL [file 13244_2025_1935_MOESM1_ESM.pdf]
